# Efficacy and safety of transcutaneous electrical acupoint stimulation to assist bowel cleansing: study protocol for a single-center, single-blind, randomized controlled trial

**DOI:** 10.3389/fmed.2026.1812594

**Published:** 2026-04-24

**Authors:** Yuanzhen Bai, Na Liu, Cheng Ye, Haifeng Jin, Yi Xu, Haibiao Bao, Liang Huang, Xue Ying, Bin Lv, Meng Li

**Affiliations:** 1 Department of Gastroenterology, The First Affliated Hospital of Zhejiang Chinese Medical University (Zhejiang Provincial Hospital of Traditional Chinese Medicine), Hangzhou, China; 2 Key Laboratory of Digestive Pathophysiology of Zhejiang Province, Hangzhou, China

**Keywords:** bowel preparation, colonoscopy, polyethylene glycol electrolyte, protocol, transcutaneous electrical acupoint stimulation

## Abstract

**Introduction:**

High-quality bowel preparation is a crucial prerequisite for ensuring the effectiveness of colonoscopy. Although polyethylene glycol (PEG) remains the first-line cleansing regimen recommended by clinical guidelines, its practical application still faces challenges in achieving adequate bowel preparation rates. Transcutaneous electrical acupoint stimulation (TEAS) shows promise in enhancing intestinal motility through gut-brain-microbiota modulation. However, its role in the field of intestinal cleansing remains unclear.

**Methods and analysis:**

This randomized controlled trial will enroll 404 patients undergoing elective colonoscopy. The participants will be randomly allocated to either the TEAS group or the sham-TEAS group, in a 1:1 ratio. TEAS was applied to stimulate the *Zusanli* (ST36) and *Neiguan* (PC6) acupoints using a clinically validated stimulation mode, administered concomitantly with PEG in a combined treatment protocol. The primary outcome measure was the rate of adequate bowel preparation, evaluated with the Boston Bowel Preparation Scale (BBPS). Secondary endpoints encompass colonoscope insertion and withdrawal times, cecal intubation rate (CIR), adenoma detection rate (ADR), first defecation time, total defecation frequency, final stool characteristics, adverse event incidence, and satisfaction.

**Discussion:**

By bridging traditional acupoint therapy with quantifiable neurogastroenterological outcomes, this study aims to establish TEAS as a scalable adjunct in bowel preparation protocols.

**Trial registration:**

International Traditional Medicine Clinical Trial Registry, ITMCTR2025001121. Registered on April 30, 2025, http://itmctr.ccebtcm.org.cn/.

## Introduction

Colorectal cancer (CRC) is the most commonly diagnosed gastrointestinal (GI) cancer, representing 1.8 million cases and 881,000 deaths globally, and constituting one in 10 cancer cases and deaths (1). Epidemiological data indicate a strong positive correlation between CRC incidence and increasing levels of economic development (2). Advanced adenomas are the most well-known precursor lesions of colorectal carcinoma (3). The early identification and resection of these lesions through screening programs have been associated with a reduction in CRC incidence and mortality rates (4, 5). A variety of screening methodologies are currently accessible, broadly categorized into stool-based tests, such as the guaiac-based fecal occult blood test and fecal immunochemical test, and direct visualization techniques, including sigmoidoscopy and colonoscopy (6, 7). Colonoscopy is used both as a primary screening tool and a confirmatory diagnostic procedure following a positive result from non-invasive tests. When comparing the performance of a single screening round, endoscopic techniques generally yielded higher detection rates of advanced neoplasia than one-time stool-based blood tests (8–10).

High-quality bowel preparation is a critical determinant of polyp and early-stage tumor detection rates during colonoscopy (11). Inadequate bowel cleansing has been associated with missed lesions, prolonged procedure duration, and a potential increase in complication rates (12–15). Current guidelines consistently recommend polyethylene glycol (PEG) as the first-line bowel preparation agent, which achieves reliable cleansing through an osmotic laxative mechanism (16). However, PEG regimens still exhibit clinical limitations, most notably suboptimal bowel cleansing efficacy. Data indicate that patients receiving conventional protocols continue to have a relatively high incidence of residual bowel content, with adequate bowel preparation rates (Boston Bowel Preparation Scale, BBPS ≥6/Ottawa Bowel Preparation Quality Scale, OBPQS ≤7) ranging from 53.1–82.2% using standardized scoring systems (17–21). Various strategies have been explored to address this challenge, including modified PEG regimens (e.g., low-volume split-dose administration), combination with prokinetic agents, or adjunctive use of stimulant laxatives like sodium picosulfate (22–25). However, these methods remain suboptimal due to variable cleansing efficacy, risks of electrolyte disturbances, and potential drug interactions. Consequently, developing non-pharmacological strategies to enhance bowel cleansing efficacy, mitigate laxative-induced gastrointestinal adverse effects, and improve treatment adherence represents a critical research direction in optimizing bowel preparation protocols.

Transcutaneous electrical acupoint stimulation (TEAS), a non-invasive stimulation therapy that combines the advantages of acupuncture and transcutaneous electrical nerve stimulation, has emerged as a promising therapeutic approach in clinical practice (26). As suggested by previous studies, TEAS has been shown to promote recovery of bowel function through modulation of the brain-gut axis (27–30). Recent clinical studies further demonstrate its therapeutic effects in functional gastrointestinal disorders (e.g., functional dyspepsia and chronic constipation), showing significant symptom relief and motility enhancement, potentially mediated by peripheral nerve activity regulation (30, 31). Additional study has confirmed that by enhancing parasympathetic activity, TEAS facilitates high-amplitude propagated contractions, which are critically required for efficient colonic propulsion (32). These mechanisms provide potential scientific support for TEAS in optimizing bowel preparation quality and alleviating the discomfort associated with the bowel preparation process.

To address the critical gap in evidence regarding TEAS for bowel preparation, we conducted a randomized, placebo-controlled clinical trial. This study hypothesizes that TEAS could optimize the quality of bowel preparation and alleviates bowel preparation-related symptoms, offering a potential adjunct to standard pharmacological regimens.

## Methods and analysis

### Trial design

**Figure 1:**
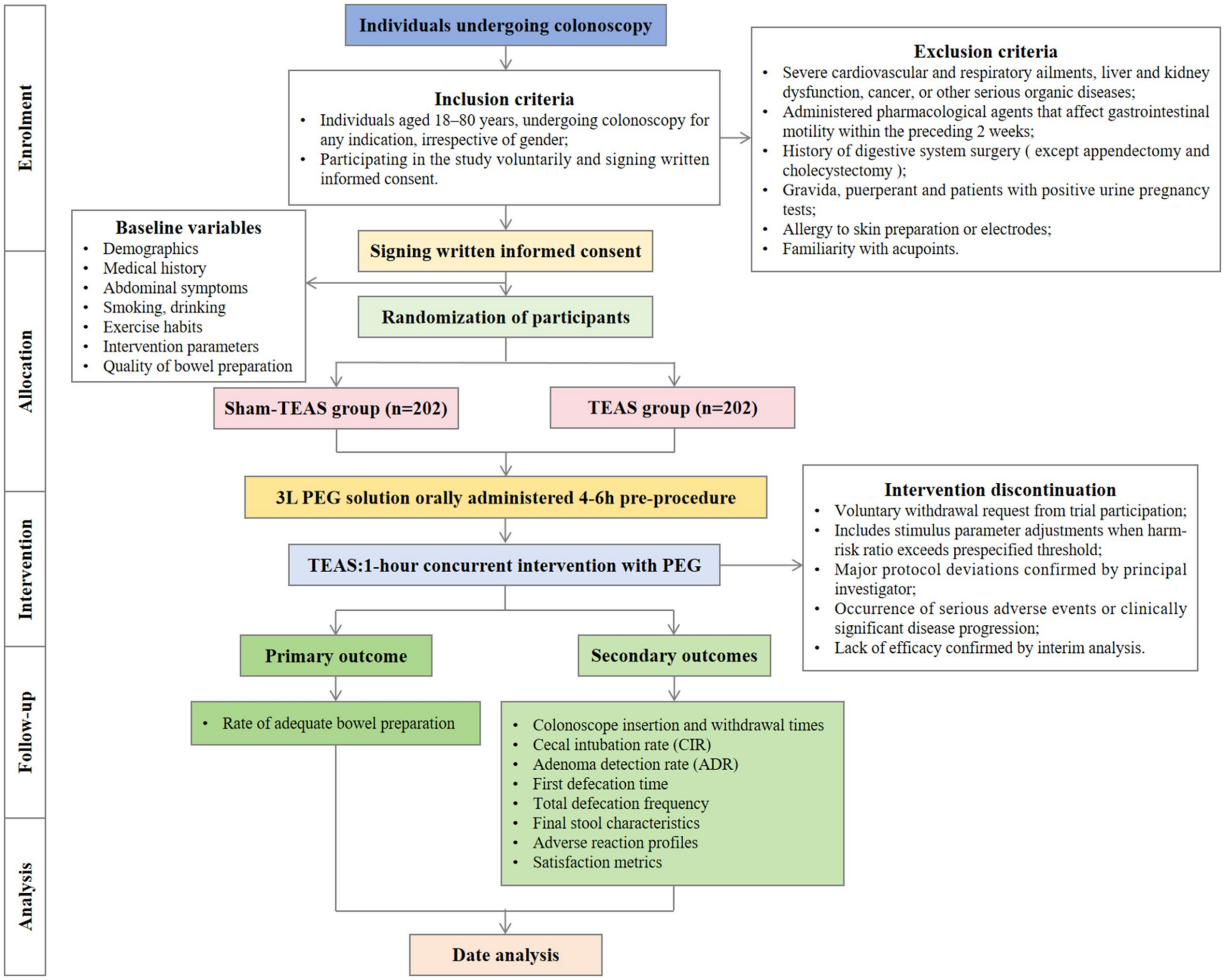
Consolidated standards of reporting trials (CONSORT) diagram for this trail.

This trial is designed as a randomised, controlled, single-blinded single-center superiority trial with a primary endpoint of the rate of adequate bowel preparation measured by the BBPS score (33). The study will be conducted at Zhejiang Provincial Hospital of Traditional Chinese Medicine in China. A total of 404 participants will be randomly allocated to either the TEAS group (Group T, *n* = 202) or the sham-TEAS group (Group S, *n* = 202) in a 1:1 ratio. To ensure objective and accurate evaluation, all study-related endoscopic procedures were performed by senior endoscopists. The research protocol adheres to the Standard Protocol Items: Recommendations for Interventional Trials (SPIRIT) guidelines (34). Table 1 presents a succinct timeline detailing the study visits, enrolment process, interventions, and assessments performed on participants. The flowchart is illustrated in Figure 1.

**Table 1:** Standard protocol items: recommendations for interventional trials (SPIRIT) schedule for enrollment, interventions, and assessments.

	Study period					
	Enrolment	Allocation	Post-allocation			Close-out
Timepoint	−24 h	0	1 h	3 h	6 h	24 h
Enrolment						
Eligibility screen	×					
Informed consent	×					
Allocation		×				
Interventions						
Sham-TEAS group			×			
TEAS group			×			
Assessments						
Baseline variables	×	×				
Quality of bowel preparation (BBPS)				×		
Rate of adequate bowel preparation				×		
Colonoscope insertion time				×		
Colonoscope withdrawal time				×		
Cecal intubation rate (CIR)				×		
Adenoma detection rate (ADR)				×		
First defecation time			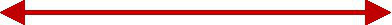			
Total defecation frequency			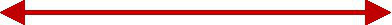			
Final stool characteristics			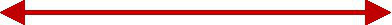			
Adverse reaction profiles			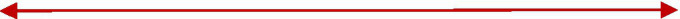			
Satisfaction metrics			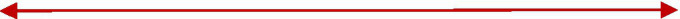			

### Eligibility criteria

#### Inclusion criteria

Patients eligible for the trial must comply with all of the following at randomization:

- Individuals aged 18–80 years, undergoing colonoscopy for any indication, irrespective of gender;
- Participating in the study voluntarily and signing written informed consent.

#### Exclusion criteria

- Severe cardiovascular and respiratory ailments, liver and kidney dysfunction, cancer, or other serious organic diseases;
- Administered pharmacological agents that affect gastrointestinal motility within the preceding 2 weeks;
- History of digestive system surgery (except appendectomy and cholecystectomy);
- Gravida, puerperant and patients with positive urine pregnancy tests;
- Allergy to skin preparation or electrodes;
- Familiarity with acupoints.

### Sample size

The sample size was calculated using a two-sample proportion test, with the following parameters: a two-sided significance level (*α*) of 0.05, a statistical power of 90% (*β* = 0.1), an expected qualified preparation rate of 70% for the sham TEAS group, and 85% for the active TEAS group. The calculated minimum evaluable sample size was 161 per group. Accounting for a 20% dropout rate, a total of 404 subjects were planned, with 202 subjects allocated to each group.

### Recruitment

This prospective study will recruit 404 adult participants scheduled for colonoscopy procedures across clinical indications at Zhejiang Provincial Hospital of Traditional Chinese Medicine. Enrollment is planned to commence in May 2025, with anticipated completion by June2026. To ensure protocol standardization, the study cohort was strictly limited to inpatients requiring scheduled colonoscopy under controlled clinical supervision. Recruitment methods mainly take place within hospitals, such as through clinician referrals and posters displayed in outpatient clinics. All recruitment activities will adhere to ethical standards, ensuring compliance with the principles of the Declaration of Helsinki (35).

### Randomization and blinding

This single-blinded trial implemented CONSORT-compliant randomization and blinding protocols (36), and subjects were assigned to the TEAS group and the sham-TEAS group in a 1:1 ratio. To ensure assessment objectivity, screening and outcome assessors remained blinded to treatment allocation status throughout the study period. All recordings were deidentified by removing all patient identifiers and temporal markers to eliminate assessment bias. The TEAS-administering acupuncturists maintained awareness of treatment allocation while remaining blinded to study outcomes. A computer-generated block randomization system with concealed allocation utilized numbered, sealed envelopes containing group assignments, secured by ward nurses until post-allocation unblinding. Primary endpoints were evaluated by two gastroenterologists independently conducted blinded assessments by reviewing endoscopic videos. A standardized operational protocol (fixed electrode stimulation duration) was implemented for double-blind control, while enabling dynamic adjustment of stimulation intensity based on individual tolerance thresholds. A blinding assessment will be conducted after colonoscopy completion and before unblinding, using a standardized questionnaire asking patients: “Which treatment do you think you received? (A. Active TEAS; B. Sham TEAS; C. Uncertain).” The success of blinding will be quantified by calculating the Bang Blinding Index.

### Interventions

#### Bowel preparation

According to the standard bowel preparation regimen recommended by the Chinese Bowel Preparation Guidelines (37), enrolled subjects orally administered a single 3 L dose of polyethylene glycol electrolyte solution (Jiangxi Hengkang Pharmaceutical Co., Ltd., Lot No. H20020031) as a one-time intake on the examination day, along with initiation of a low-residue diet 24 h prior to colonoscopy. All patients must consume PEG solution 4 to 6 h prior to the endoscopy, drinking 250 mL every 10 min and completing it within a 2-h timeframe. To ensure protocol adherence, two groups received standardized verbal bowel preparation instructions from researchers during colonoscopy scheduling, complemented by detailed written materials outlining preparation steps to minimize comprehension and compliance. Patients in different study groups were allocated to spatially isolated designated wards during TEAS treatment, with strict restrictions on cross-ward activities.

#### Colonoscopy

All colonoscopies were conducted with the CF-H290l high-resolution adult video colonoscope (Olympus, Tokyo, Japan) by senior endoscopists (≥5 years experience). The endoscopists were blinded to the study’s hypotheses, objectives and patient randomization. Sedation for the colonoscopy was administered using a combination of alfentanil and propofol.

#### TEAS and sham-TEAS

**Figure 2:**
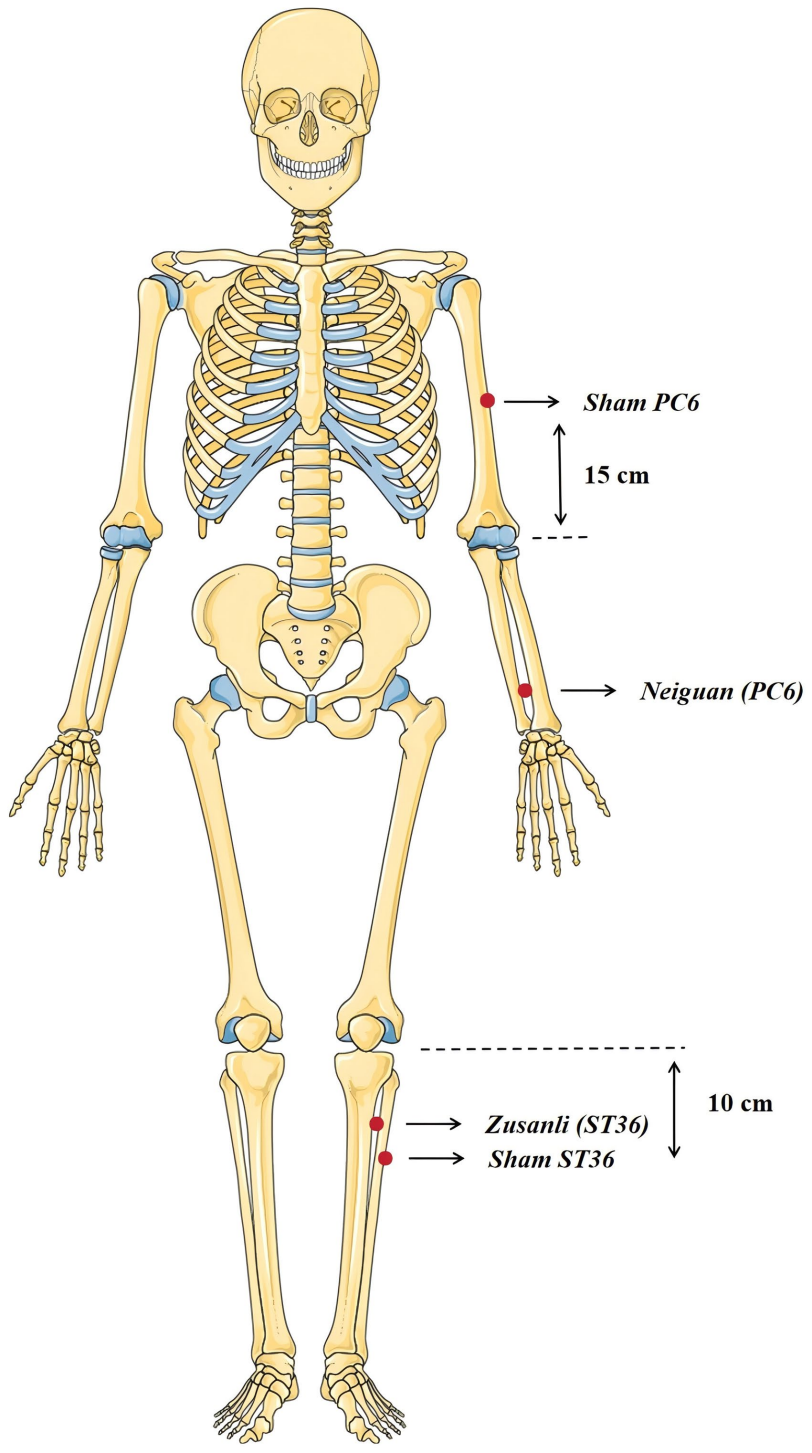
Location of acupoints and sham-acupoints. Graphical elements by Servier Medical Art (https://smart.servier.com), licensed under CC BY 4.0 (https://creativecommons.org/licenses/by/4.0/).

**Figure 3:**
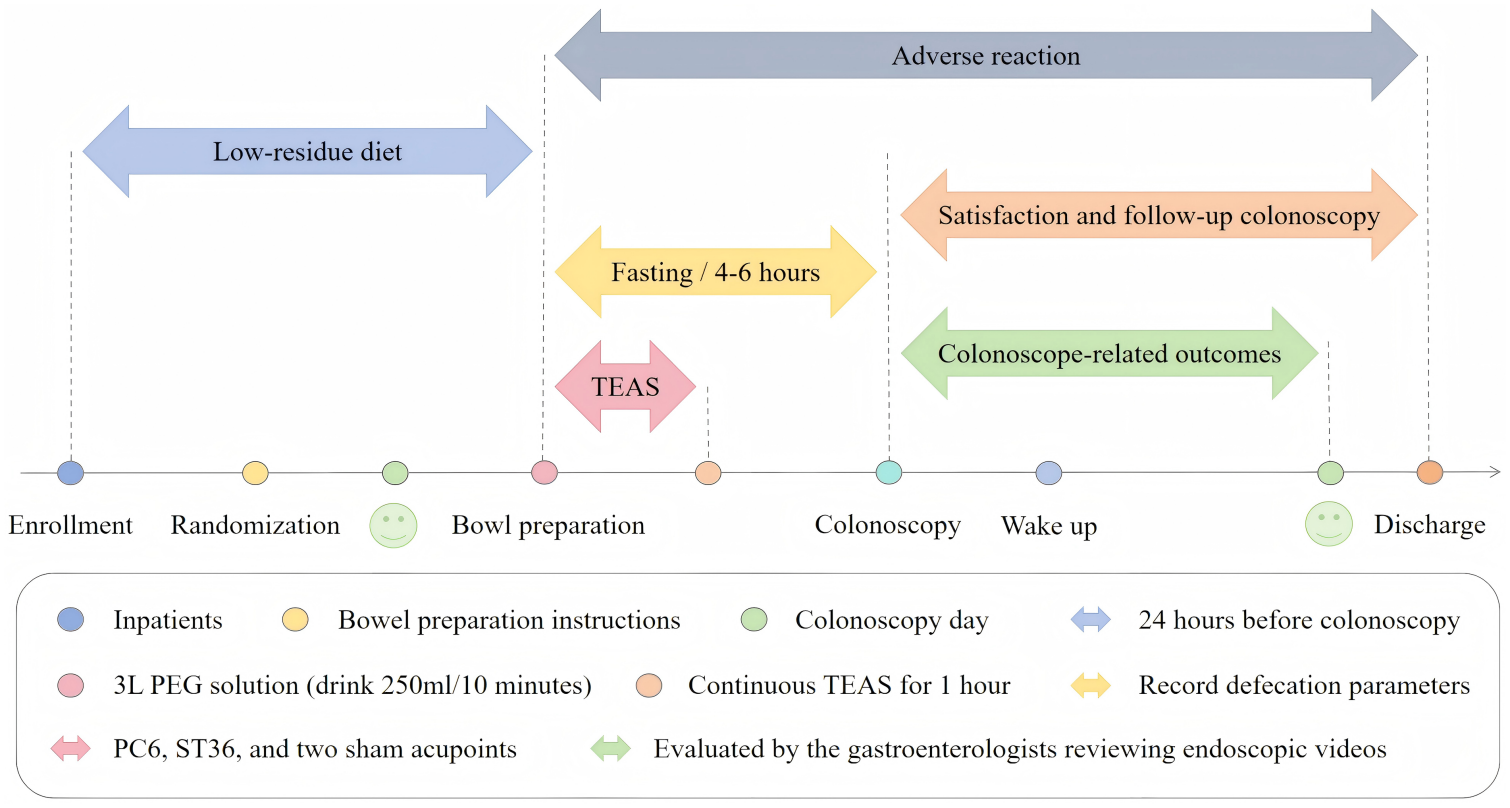
The administration of TEAS treatment protocol.

TEAS Protocol: The TEAS intervention protocol employed an acupoint combination targeting *Neiguan* (PC6) and *Zusanli* (ST36) (see Figure 2 for anatomical landmarks). PC6 acupoint is located on the anterior forearm, 2 cun proximal to the transverse wrist crease, between the tendons of the palmaris longus and flexor carpi radialis muscles. ST36 acupoint is situated on the anterolateral aspect of the leg, 3 cun inferior to the lower border of the patella, one finger-breadth lateral to the anterior crest of the tibia. Pre-intervention anatomical validation by acupuncturists (<3 mm positioning tolerance). The stimulation system comprised hydrogel self-adhesive electrodes (diameter: 20 mm) interfaced with a transcutaneous acupoint electrical stimulator (SNM-FDCM01, Ningbo Maida Medical Instrument Company, Ningbo, China). The intervention period overlapped with the PEG administration period and lasted 1 h from the first dose of PEG ingestion (Figure 3). The stimulation parameters were set as follows: a train on-time of 2 s and off-time of 3 s, pulse width of 0.5 ms, pulse frequency of 25 Hz, and amplitude of 2–10 mA (at the maximum level tolerated by the subject).

Sham-TEAS Protocol: Sham acupoint stimulation was performed using the same devices to provide identical parameters as TEAS, with sham points positioned outside the anatomical course of relevant meridians: the sham-point of PC6 is approximately 15 cm up to the elbow joint and lateral to the real PC6 acupoint and the sham-point of ST36 is approximately 10 cm down to the knee and lateral to the real ST36 acupoint. The temporal schedule and ancillary procedures (electrode placement duration, PEG coordination) mirrored the intervention group.

#### Rationale for parameter selection

PC6 (Neiguan) and ST36 (Zusanli) are classic acupoints for gastrointestinal disorders. ST36 is the Lower He-Sea point of the stomach, and PC6 connects to the Yin Link Vessel. Modern studies confirm that PC6 electroacupuncture promotes gastric emptying and small intestinal transit via vagal afferent activation (38) and reduces postoperative nausea and vomiting (39). ST36 stimulation promotes distal colonic peristalsis and accelerates colonic transit through the sacral parasympathetic efferent pathway (40). Although abdominal surface stimulation may be more directly relevant to promoting bowel motility (41), it was not adopted due to potential provocation of PEG-related abdominal pain, low evidence level, and unclear optimal parameters. In contrast, limb-based PC6 and ST36 are easy to secure and do not interfere with bowel preparation. Regarding stimulation parameters (42, 43): 25 Hz is a mid-frequency dense-disperse wave that effectively activates vagal efferent fibers to promote colonic motility. A pulse width of 0.5 ms is standard for transcutaneous electrical nerve stimulation, balancing skin penetration depth and fiber selectivity. The 1-h duration overlaps the critical period of intestinal filling and enhanced peristalsis following PEG ingestion. Intensity is individualized to patient tolerance from 2 to 10 mA, ensuring efficacy without causing pain or skin injury.

#### Standardization of intervention implementation

To ensure consistency in intervention implementation across different operators, a standardized training and quality control system was established for this study. All operators uniformly completed theoretical, video-based, and manikin simulation training. They were required to achieve 100% accuracy in assessments of acupoint localization, electrode placement, and parameter settings (25 Hz, 0.5 ms, 2–10 mA) before being permitted to participate. During the study period, retraining sessions were conducted every two weeks, 10% of operational records were randomly audited, and inter-operator consistency was regularly evaluated.

### Outcomes

#### Primary outcome

The primary outcome measure was the rate of adequate bowel preparation, which was assessed by two gastroenterologists through independent review of endoscopic videos using the Boston Bowel Preparation Scale (BBPS) (33). The BBPS was applied to each of the three segments of the colon (right, including cecum and ascending colon; transverse, including hepatic and splenic flexures; and left, including descending colon, sigmoid and rectum). Each colon region received a “segment score” ranging from 0 to 3, and these scores were summed to calculate a total BBPS score (0–9) (refer to Appendix). Adequate bowel preparation was defined as a total BBPS score ≥6 with each colon segment scoring ≥2 after standard washing and air insufflation for luminal distension during colonoscopy (13, 44). Endoscopists maintained blinding to group allocation throughout the trial. All endoscopic imaging was independently evaluated by two gastroenterologists, with final diagnoses established through consensus deliberation and unresolved discrepancies resolved by a third-party senior specialist.

#### Secondary outcomes

- Defecation parameters

Following TEAS/sham-TEAS interventions, patients documented their defecation parameters in the record sheets, including first defecation time, total defecation frequency, and final stool consistency.

- Colonoscope outcomes

The evaluated parameters encompassed four key indicators: insertion time, withdrawal time, cecal intubation rate (CIR), and adenoma detection rate (ADR). The insertion phase was defined as commencing with initial rectal visualization and concluding when the endoscope tip traversed the ileocecal valve into the cecal caput. Withdrawal time represents the duration from cecum to anal canal during meticulous mucosal inspection in screening or diagnostic procedures without therapeutic interventions, with a minimum 6-min benchmark indicating thorough pathological evaluation. CIR, which reflects procedural completeness through comprehensive cecal visualization including anatomical landmarks, must be maintained at ≥95% to ensure examination adequacy. ADR calculated as the percentage of colonoscopies with at least one adenoma identified.

- Adverse reaction

Research staff documented all adverse reactions occurring during the bowel preparation process and post-colonoscopy, including nausea, vomiting, abdominal pain, distension, headache, and dizziness (refer to Appendix). The incidence rate of adverse reactions was calculated as (number of cases with adverse reactions)/(total number of cases).

- Satisfaction and follow-up colonoscopy

At the conclusion of the treatment period, all participants were surveyed regarding their satisfaction with bowel preparation and their willingness to have a follow-up colonoscopy. The details are presented in Appendix.

### Data collection and management

This study will systematically collect baseline data (demographics, medical history, abdominal symptoms assessments [using the Gastrointestinal Symptom Rating Scale (45), GSRS], intervention parameters (electrostimulation settings, acupoint localization, compliance), efficacy endpoints, and safety profiles. Data will be recorded via electronic date capture and paper-based case report forms (CRFs) during screening, intervention, and follow-up phases, with real-time entry and logic verification. Two individuals will independently enter and verify the data for error correction, and participant information will be anonymized. The Ethics Committee will conduct random audits for quality control to ensure the authenticity and traceability of the data.

### Statistical analysis

All statistical analyses were performed using SPSS 29.0 and GraphPad Prism 10.4, adhering to the intention-to-treat (ITT) principle. Continuous variables were tested for normality using the Shapiro–Wilk test; normally distributed data were expressed as mean ± standard deviation, and non-normally distributed data as median [interquartile range]. Categorical variables were presented as frequencies and percentages. For the primary outcome (rate of adequate bowel preparation), a multivariable logistic regression model was employed to adjust for baseline covariates. Missing data were handled using Multiple Imputation by Chained Equations (MICE); 20 imputed datasets were generated, and the imputation model included all baseline variables, the grouping variable, and the primary outcome measure. To control the type I error rate for multiple secondary outcomes, a hierarchical strategy was adopted. The primary outcome was tested at *α* = 0.05. Among secondary outcomes, colonoscopy indicators were designated as key secondary endpoints and were corrected using the Holm-Bonferroni method. For these endpoints, insertion and withdrawal times (continuous variables) were compared using independent *t*-tests or Mann–Whitney *U* tests as appropriate, and cecal intubation and adenoma detection rates (categorical variables) were compared using Chi-square or Fisher’s exact tests. The remaining secondary outcomes, including bowel movement parameters, patient satisfaction scores, and adverse reactions, were treated as exploratory analyses; no multiplicity correction was applied, and *p*-values are presented for descriptive reference only. Safety profiles were described by comparing the incidence and severity of adverse events (Chi-square test) and patient satisfaction (Wilcoxon rank-sum test). Protocol deviations were addressed by per-protocol sensitivity analyses to verify the robustness of the results. A two-sided *p* < 0.05 was considered statistically significant.

### Safety assessment

All enrolled patients were included in the safety assessment. Safety outcomes encompassed predefined adverse events (including ear skin rash, dizziness, palpitation, and intolerable diarrhea requiring intervention) during treatment and follow-up phases. All safety-related data were meticulously documented for safety findings.

## Discussion

This study proposes a novel therapeutic paradigm integrating traditional meridian theory with contemporary neurogastroenterology, hypothesizing that TEAS at gastrointestinal-associated acupoints (ST36 and PC6) may optimize bowel preparation efficacy while mitigating laxative-related discomfort. To our knowledge, this randomized controlled trial represents the first clinical investigation employing TEAS as an adjunctive modality for bowel preparation in colonoscopy, constituting an original contribution to endoscopic preparation research.

Despite established protocols, suboptimal bowel preparation remains a persistent challenge in gastrointestinal endoscopy, with multicenter studies revealing inadequate cleansing rates ranging from 20 to 40% across diverse populations (13, 14, 46, 47). The predominant use of PEG solutions, while effective, is marred by significant limitations: a multicentre randomized controlled trial (*n* = 422) documented 37% gastrointestinal intolerance rates, including nausea, bloating, abdominal pain/cramps and anal irritation, contributing to 17.8% patient refusal to-repeat the same preparation for subsequent colonoscopies (48). More critically, the ability to detect adenomas and advanced adenomas is significantly hampered by inadequate bowel preparation. A systematic review and meta-analysis of 11 studies (more than 55,000 colonoscopies) identified inadequate preparation as the primary contributor of missed neoplastic lesions, with ADR decreasing by 5% in poorly prepared segments (49). Yang et al. (50) revealed that 15–37% of screening candidates experience anxiety regarding bowel preparation regimens, which consequently compromises strict adherence to prescribed purgative protocols and dietary restrictions. In conclusion, such preparation failures impose substantial clinical and economic burdens while posing risks and inconveniences for patients (51). Therefore, high-quality bowel preparation serves as an indispensable prerequisite for enhancing ADR and decreasing procedure-related complication rates, while ensuring screening efficacy and patient safety.

In order to solve these problems, several bowel preparation agents, including sodium picosulfate-magnesium citrate, ascorbate-enriched PEG, sodium phosphate solution, and oral sulfate solution, were developed; however, with the exception of oral sulfate solution, none achieved the 90% quality benchmark (52). In addition, these hyperosmotic and stimulant agents have been reported to increase risks such as colonic ischemia, renal impairment, and electrolyte disturbance (11). A clinical study involving 271 elderly constipated patients demonstrated that abdominal vibration (physical stimulation) combined with walking exercise achieved an adequate bowel preparation rate of 92.2% (53). For outpatients, interval walking exercise implemented after PEG administration prior to colonoscopy also proved effective in improving bowel preparation quality (54). The limitation lies in the population-specific efficacy of this intervention, showing more pronounced effects in elderly patients and young, non-obese, ambulatory patients without a history of abdominal or gynecological surgery (53, 54). Dietary interventions including clear liquid diets and low-residue diets (LRD), are also widely adopted for bowel preparation (55, 56); however, notable limitations such as the time-consuming, patient discomfort, and lack of convenience hinder the application of these methods (14). Some authors even argue that LRD may reduce bowel motility and prolong intestinal transit, resulting in effects contrary to the intended purpose, particularly in constipated patients (57). In summary, it is necessary to explore an adjuvant regimen for bowel preparation characterized by broad applicability across populations, favorable safety profile, high patient compliance, and superior cost-effectiveness.

Acupuncture is a nonpharmaceutical technique that originated in China and has been used for over 3,000 years. TEAS is a modification of traditional acupuncture (58–60), which combines the advantages of acupuncture and percutaneous electrical nerve stimulation. Unlike traditional acupuncture requiring skin penetration, this transcutaneous approach exhibits notable application prospects via its non-invasive nature, user-friendly operation, and portability (27, 61–63), while concurrently reducing infection risks and improving patient compliance (64). In recent years, a growing number of investigations have put a spotlight on the therapeutic effects of TEAS in intestinal dysmotility disorders. Clinical trials demonstrated that ST36 acupoint stimulation effectively enhances spontaneous bowel movements, alleviates constipation symptoms, and improves quality of life (65), while PC6 application demonstrates marked efficacy in reducing postoperative nausea and vomiting incidence within 24 h (66). While transabdominal or abdominal surface stimulation is theoretically more direct, limb acupoint TEAS is a superior adjunctive choice based on current evidence and operational feasibility. This study implemented a standardized stimulation parameter protocol (0.5-ms pulse width, 25 Hz frequency, and 2–10 mA amplitude) (42, 43, 67), developed based on prior clinical trial evidence to ensure intervention stability and experimental reproducibility. This study recruited only inpatients, which may limit the generalizability of our findings to the broader outpatient colonoscopy population. The inpatient setting was chosen to ensure protocol adherence and intervention fidelity, but future multicenter, pragmatic trials in outpatient settings are needed to validate the real-world applicability of TEAS as an adjunct to bowel preparation. Additionally, although we standardized endoscopist experience and withdrawal time, ADR remains influenced by multiple operator-dependent factors, and residual confounding cannot be fully excluded.

This protocol employs validated stimulation parameters (25 Hz frequency, 60-min sessions) combined with standardized laxative regimens, aiming to establish TEAS as a safe strategy for enhacing bowel preparation and alleviating adverse effects (e.g., bloating and nausea).

## Appendix

### The Boston bowel preparation scale

BBPS was applied to each of the three broad regions of the colon: the right colon (including the cecum and ascending colon), the transverse colon (including the hepatic and splenic flexures), and the left colon (including the descending colon, sigmoid colon, and rectum). The points are assigned as follows:

0 = Unprepared colon segment with mucosa not seen due to solid stool that cannot be cleared.
1 = Portion of mucosa of the colon segment seen, but other areas of the colon segment not well seen due to staining, residual stool and/or opaque liquid.
2 = Minor amount of residual staining, small fragments of stool and/or opaque liquid, but mucosa of colon segment seen well.
3 = Entire mucosa of colon segment seen well with no residual staining, small fragments of stool or opaque liquid.

### Adverse reaction

Adverse reaction associated with bowel preparation and colonoscopy procedures—including nausea, vomiting, abdominal pain, bloating, headache, and dizziness—were prospectively categorized by blinded independent assessors.

1) Transient symptoms requiring no intervention, with preserved procedure tolerability;
2) Symptom-driven procedural pauses (<5 min) without compromising examination completion;
3) Intolerable symptoms necessitating premature procedure termination;

### Patient satisfaction

Overall satisfaction with bowel preparation:

0 = A very poor experience with a very painful process and many adverse effects;
5 = The average level; there are some side effects, but acceptable;
10 = The examination has no adverse effects;

Readiness to undergo follow-up colonoscopy

0 = Great reluctance to undergo the examination again;
5 = If necessary, you reluctantly accept to take that;
10 = Willingness to undergo the examination again if necessary.
